# A Rare Presentation of a Large Pleomorphic Rhabdomyosarcoma of the Inferior Vena Cava (IVC): A Case Report

**DOI:** 10.7759/cureus.102016

**Published:** 2026-01-21

**Authors:** Kabhisha Gunasekaran, Andrew Coveney

**Affiliations:** 1 Acute Surgical Unit, Fiona Stanley Hospital, Murdoch, AUS; 2 General Surgery, Sir Charles Gairdner Hospital, Perth, AUS

**Keywords:** chemoradiation, complicated abdominal sarcoma, inferior vena cava thrombus, pleomorphic rhabdomyosarcoma, “retroperitoneum”

## Abstract

Rhabdomyosarcoma (RMS) in adults is a rare, aggressive malignant tumour of mesenchymal origin with a poor prognosis. Hematoxylin and eosin (H&E) staining showing cellular population of large, rounded epithelioid, rhabdoid, plump spindled, and bizarre multinucleate tumour giant cells, as well as immunohistochemistry showing extensive staining of the atypical tumour cells for desmin and myogenic differentiation 1 gene (MyoD1) with multifocal coexpression of myogenin, are characteristic of RMS. We present a rare and unique case of a male in his 50s who presented with a two-week history of right-sided intermittent dull groin and back pain associated with bilateral lower limb swelling, who was diagnosed with a 9 cm right retroperitoneal pleomorphic RMS involving the inferior vena cava (IVC), causing complete IVC occlusion. He underwent an extensive en bloc resection of the tumour. We also discuss the signs, symptoms, relevant investigations, and various treatment options in managing a patient with pleomorphic RMS.

## Introduction

Rhabdomyosarcoma (RMS) is classified as the most aggressive malignant tumour of mesenchymal (immature striated muscle) origin [1,2]. Compared to children and adolescents, the occurrence of RMS in adults is rare, affecting mostly extremities [2]. RMS in adults has a poor prognosis due to the aggressive nature of the tumour and poor response to adjuvant chemoradiotherapy [2]. RMS accounts for less than 1% of all solid tumour malignancies and 3% of all soft tissue sarcomas among the adult population [3]. Adult patients are more frequently diagnosed with RMS subtypes, such as pleomorphic and RMS not otherwise specified (RMS NOS), while embryonal and alveolar RMS are more commonly seen in children and the adolescent population [1-4]. RMS can have various primary anatomic sites; however, the most common sites are the head and neck region (38%) and subsequently the genitourinary region (22%) and extremities (18%), followed by other less frequent sites such as abdomen, chest, perineum, anal region, and retroperitoneum [2,3]. Interestingly, the epidemiology of primary tumour site depends on patients’ age and histological subtype, and adult RMS patients have increased chances of developing primary tumours at unfavourable anatomical regions [1,3]. This case report describes an unusual and unique case of adult right retroperitoneal pleomorphic RMS arising from a rare location, infrarenal inferior vena cava (IVC), causing complete IVC occlusion and multiple venous thrombosis, and discusses the clinical presentation, history, examination, investigation, and management of adult pleomorphic RMS. The patient had a survival duration of 11 months post an aggressive, successful en bloc resection of the IVC tumour. This case report emphasises the need for further studies that may help in contributing in future management of patients with RMS.

## Case presentation

**Figure 1:**
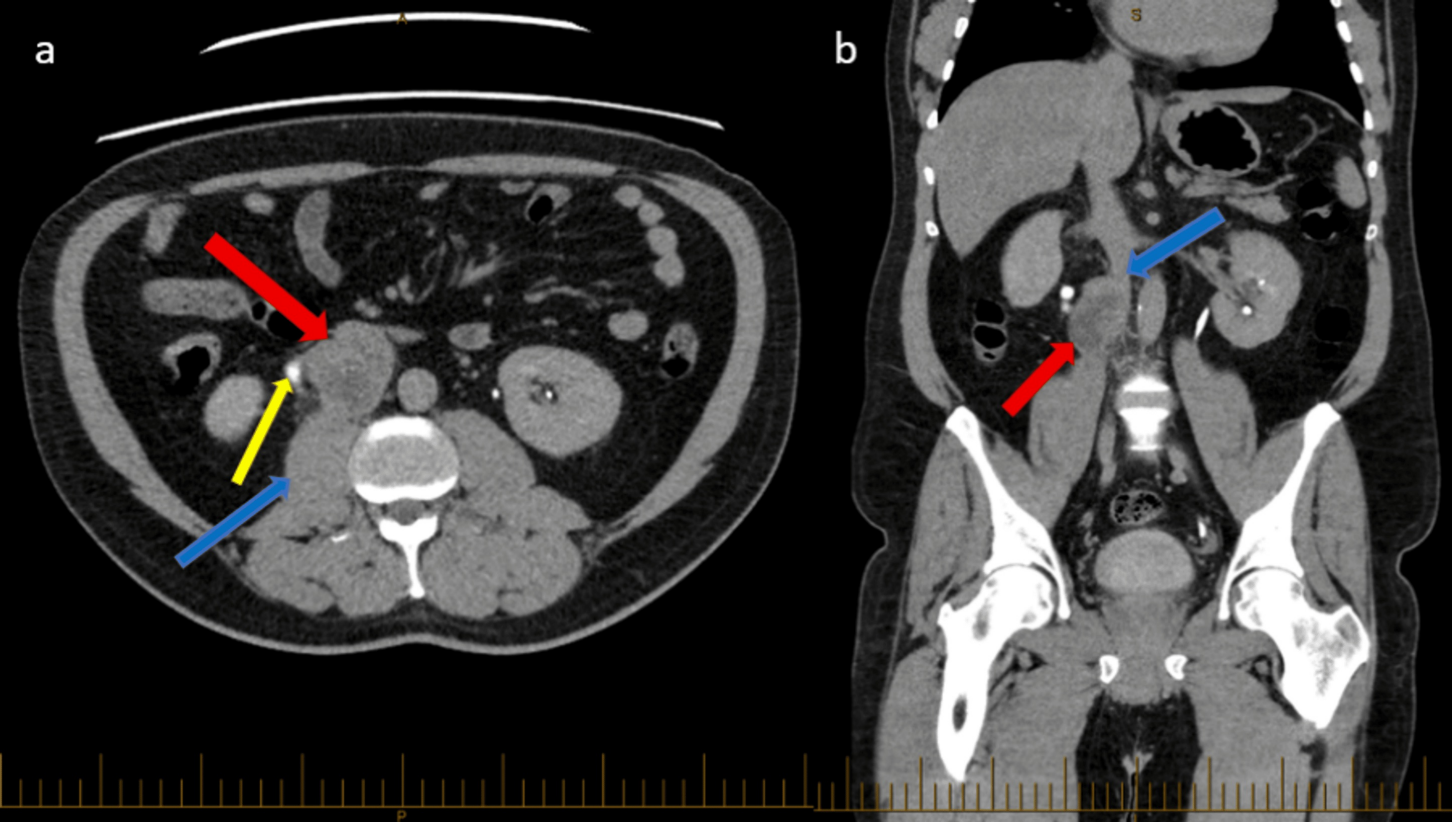
CT IVP delayed phase pre-operatively CT: computed tomography; IVP: intravenous pyelogram; IVC: inferior vena cava (a) Axial image demonstrating a 53 x 39 x 60 mm lobulated mass (red arrow) along the course of the right ureter (yellow arrow) with likely local invasion of the right anterior margin of the psoas muscle (blue arrow). (b) Coronal slice demonstrating the lobulated mass (red arrow) invading the IVC lumen (blue arrow)

A man in his 50s presented with a two-week history of right-sided intermittent dull groin and back pain with associated three-day history of bilateral lower limb swelling and anterolateral thigh pain as well as fever, rigors, and reduced appetite. He initially presented to the hospital three weeks prior for haematuria and had a right ureteric stent inserted for obstructive uropathy secondary to a 6 cm right-sided heterogeneous retroperitoneal mass compressing the right ureter, causing moderate hydronephrosis and hydroureter. The mass was abutting the IVC and psoas muscle, raising the concern of IVC patency, as evident by initial imaging with computed tomography (CT) scan (Figure 1). His CT chest performed for staging showed no evidence of thoracic metastatic disease. He has gastroesophageal reflux disease (GERD) and takes esomeprazole as required. He has no known drug allergies. He is an ex-smoker, quitting 15 years prior and drinks minimal alcohol. He has no previous colonoscopies or gastroscopies. He has a family history of hypertrophic cardiomyopathy (HCM).

On presentation, he was tachycardic and had a low-grade temperature of 37.9°C. On examination, his abdomen was soft and non-tender. He demonstrated pitting oedema in bilateral lower limbs. Septic workup was done, and urinalysis was positive for blood, protein, and leukocytes. His blood markers are as shown in Table 1, which includes an elevated white cell count (WCC) of 11.27 x 10^9^/L and C-reactive protein (CRP) of 260 mg/L.

**Table 1:** Laboratory findings of the patient with normal range for reference. eGFR: estimated glomerular filtration rate; H: high; L: low

	Level	Normal range
White cell count (WCC)	11.27 x 10^9^/L H	4.00-11.00 x 10^9^/L
C-reactive protein (CRP)	260 mg/L H	<5.0 mg/L
Urea	10.4 mmol/L H	3.0-8.0 mmol/L
Creatinine	119 umol/L H	60-110 umol/L
eGFR	58 mL/min/1.73m^2 ^L	>60 mL/min/1.73m^2^

**Figure 2:**
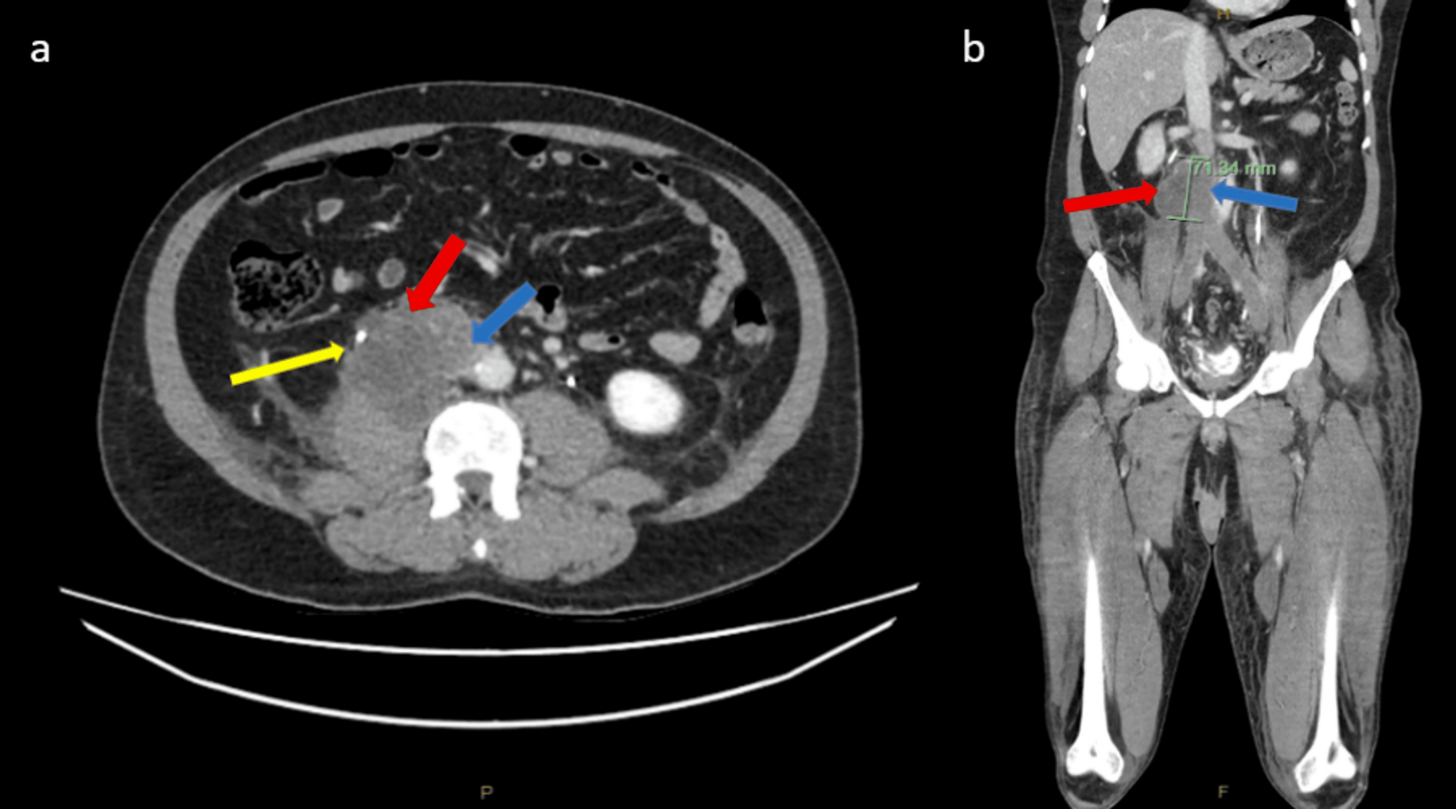
CT portal venous phase pre-operatively CT: computed tomography; IVC: inferior vena cava (a) Axial slice demonstrating an interval increase in size of the right retroperitoneal mass (red arrow) to 69 x 73 x 71 mm lying in proximity to the right ureter (yellow arrow) and IVC (blue arrow). (b) Coronal slice demonstrating the lobulated mass (red arrow) invading the IVC lumen (blue arrow)

**Figure 3:**
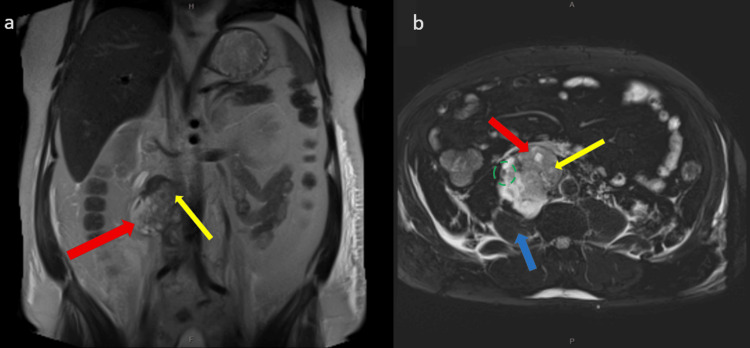
MRI T2 phase pre-operatively MRI: magnetic resonance imaging; IVC: inferior vena cava (a) Coronal slice demonstrating a right-sided malignant tumour (red arrow) with involvement of the third part of the duodenum (yellow arrow). (b) Axial slice demonstrating a lobulated mass (red arrow) invading the IVC lumen (yellow arrow) with involvement of right ureter (green dotted oval), and right psoas (blue arrow)

On presentation, he had repeat CT imaging of pulmonary angiogram (CTPA), abdomen and pelvis within one month post initial imaging, which showed an interval increase in size of the right retroperitoneal mass to 73 mm (Figure 2) and progressive obstructing thrombus within the IVC extending to the popliteal veins with no evidence of pulmonary embolism. Pre-operative magnetic resonance imaging (MRI) showed a malignant tumour invading the IVC (Figure 3) with involvement of the right ureter, right psoas, and duodenum, as well as bilateral ilio-femoral deep vein thrombosis (DVT).

**Figure 4:**
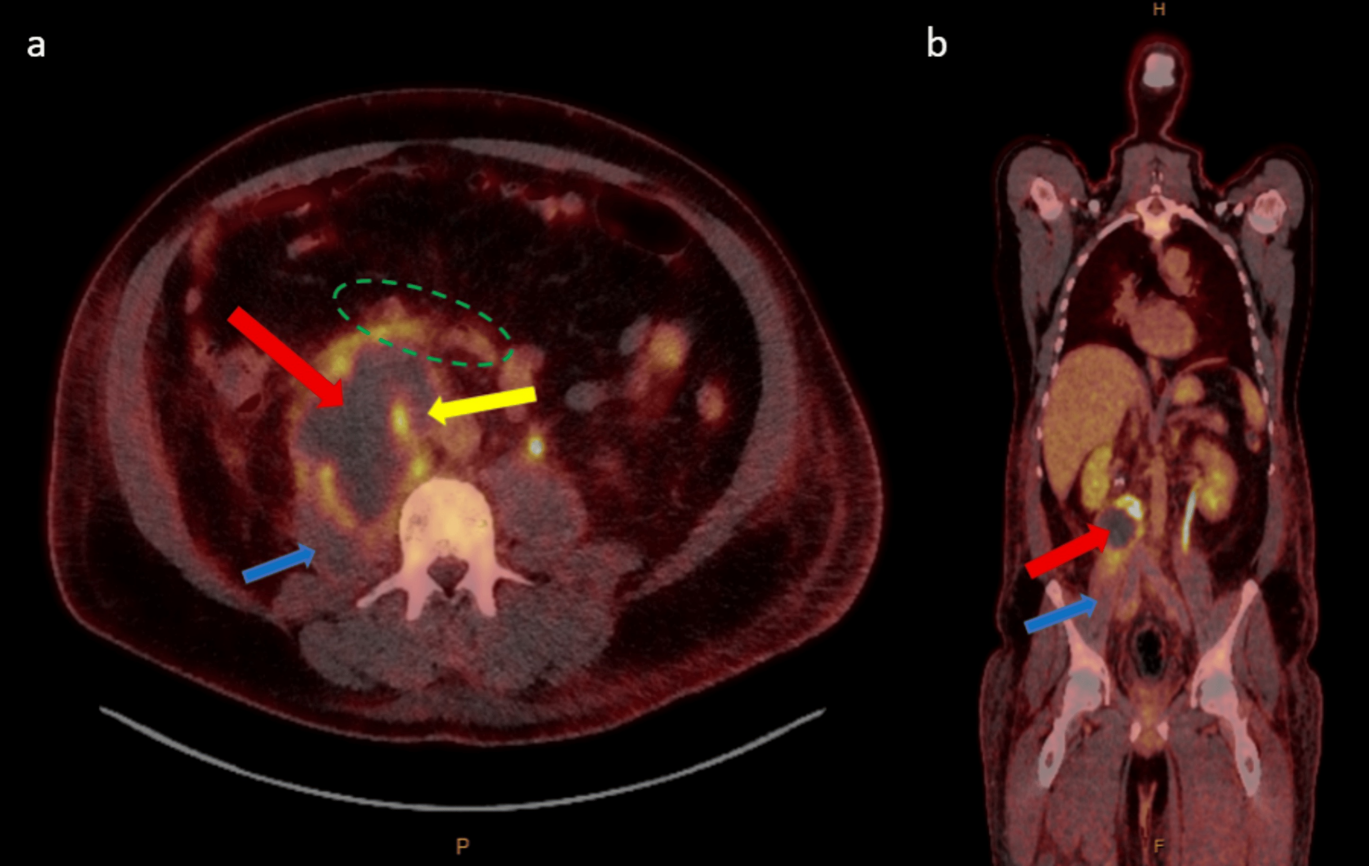
a-b showing fused PET-CT phase pre-operatively PET: positron emission tomography (PET); CT: computed tomography; IVC: inferior vena cava (a) Axial slice demonstrating a right retroperitoneal mass (red arrow) with central photopenia and peripheral moderate to high-grade increased activity extending to the right psoas muscle (blue arrow), duodenum (green dotted oval), and IVC (yellow arrow). (b) Coronal slice demonstrating right retroperitoneal mass (red arrow) with activity extending to the right psoas muscle (blue arrow)

His imaging suggested a right retroperitoneal sarcoma causing IVC compression and right-sided hydronephrosis. He did not have a pre-operative biopsy due to the acute nature of the IVC thrombus requiring anticoagulation and the need for intervention regardless of histology. His positron emission tomography (PET) scan (Figure 4) showed a right retroperitoneal mass with activity extending to the right psoas muscle posteriorly, duodenum anteriorly, and IVC superiorly, as well as no metabolically active nodal or distant metastatic disease. After a period of optimisation for surgery, he proceeded to surgery as a joint case with vascular and general surgery. During the pre-operative workup period, he was given IV heparin infusion and thromboembolic deterrent stockings (TEDS) for DVT.

**Figure 5:**
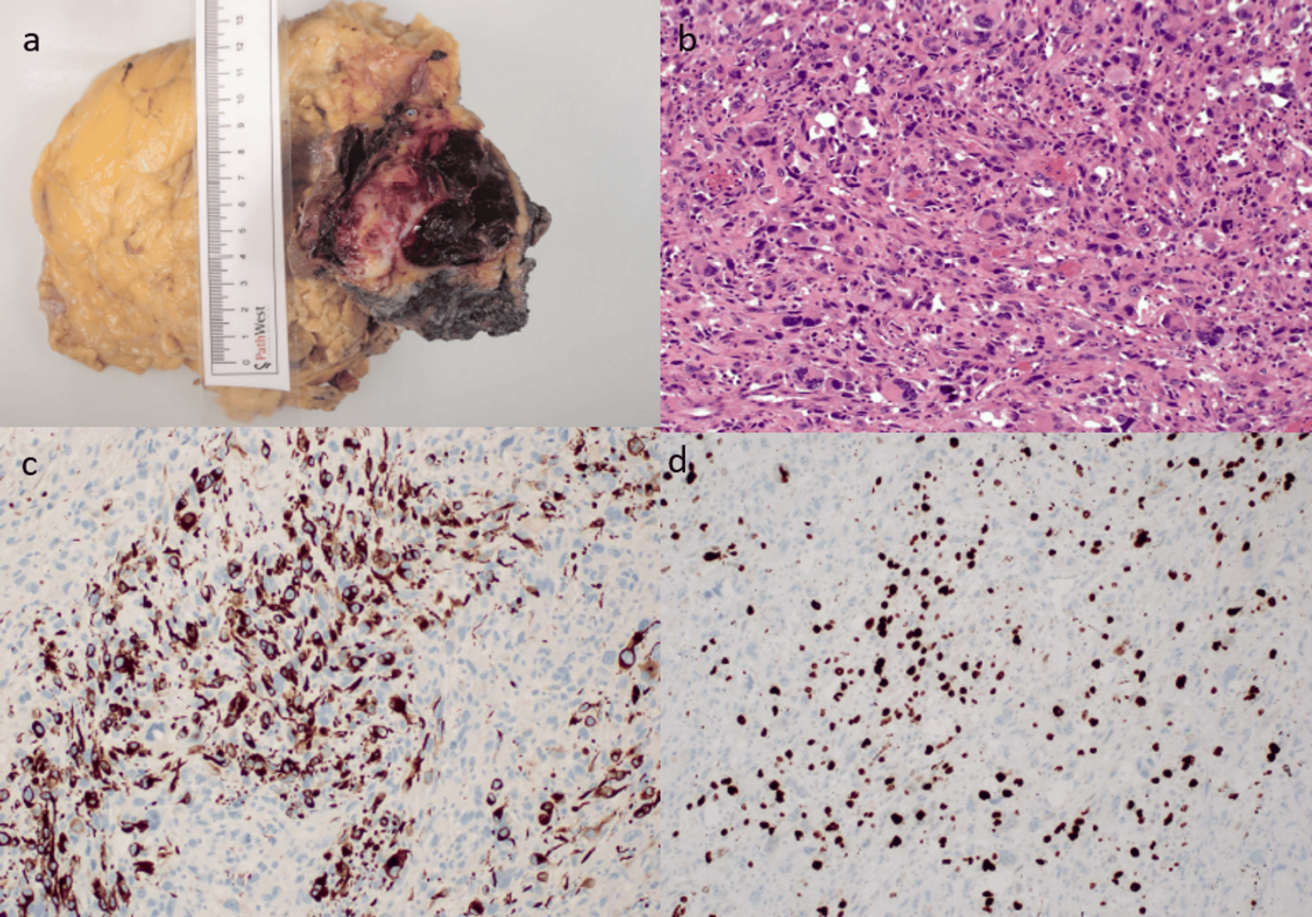
a-d shows macroscopic and microscopic findings of the tumour H&E: hematoxylin and eosin; MyoD1: myogenic differentiation 1 gene (a) Macroscopic image of the en bloc resection measuring overall dimensions 300 x 170 x 110 mm with tumour size of 90 x 60 x 60 mm. (b) H&E staining showing cellular population of large, rounded epithelioid, rhabdoid, plump spindled and bizarre multinucleate tumour giant cells occurring in sheets. (c) Immunohistochemistry showing extensive staining of the atypical tumour cells for desmin. (d) Immunohistochemistry showing extensive staining of the atypical tumour cells for MyoD1 with multifocal coexpression of myogenin

He underwent an extensive laparotomy with curative intent, which involved resection of the IVC tumour with en bloc right hemicolectomy, the inferoposterior wall of the third part of the duodenum, right anterior psoas, right nephrectomy, and right adrenalectomy. He also had bilateral ilio-femoral vein thrombectomy and IVC reconstruction with a 22 mm polytetrafluoroethylene (PTFE) tube graft. The findings confirmed an infrarenal IVC tumour invading the psoas muscle posterolaterally, right colonic mesentery anteriorly, and the third part of the duodenum superiorly. His stented right ureter was also encased by the tumour. His IVC had a palpable, hard thrombus extending above the renal veins and below into the bilateral iliac systems. The retroperitoneal tissues were very inflamed secondary to the thrombus with multiple venous collaterals. Veno-veno bypass was not performed as the IVC was already occluded for at least five days prior to the surgery, so cross-clamping the IVC did not cause significant cardiac compromise. The IVC was replaced with a PTFE tube graft after removal of the thrombus from the iliac systems. A frozen section of fat between the IVC and the aorta was sent and was negative for tumour. His post-operative histopathology showed pleomorphic RMS of the right retroperitoneum, grade 3 (Fédération Nationale des Centres de Lutte Contre le Cancer (FNCLCC)), negative for MDM2/CDK4 amplification, with full-thickness invasion of the IVC wall forming luminal tumour thrombus with luminal fibrin-thrombus at the superior margin of the IVC, likely admixed with largely necrotic tumour cells and extensive involvement of psoas muscle, but no nodal involvement. The tumour was abutting but not invading the ureteric and duodenal walls, as well as no involvement of the right kidney, right adrenal, right colon, or terminal ileum. The histopathology findings are shown in Figure 5. His estimated blood loss was 5 L, as he was anticoagulated throughout the entire operation.

Post-surgery, he required ICU admission for ionotropic support. He received IV tazocin as prophylactic cover to protect the IVC graft, multiple blood product transfusions for anaemia, and anticoagulation treatment with heparin. He was started on total parenteral nutrition (TPN) on day one post-surgery. He was extubated within 24 hours post-surgery and weaned off noradrenaline on day two post-surgery. His post-operative course was complicated by an acute kidney injury, given that he had a right nephrectomy, and the left renal vein was also ligated. This required temporary dialysis via a Hickman line. He was discharged 23 days post-surgery on warfarin for his IVC graft.

**Figure 6:**
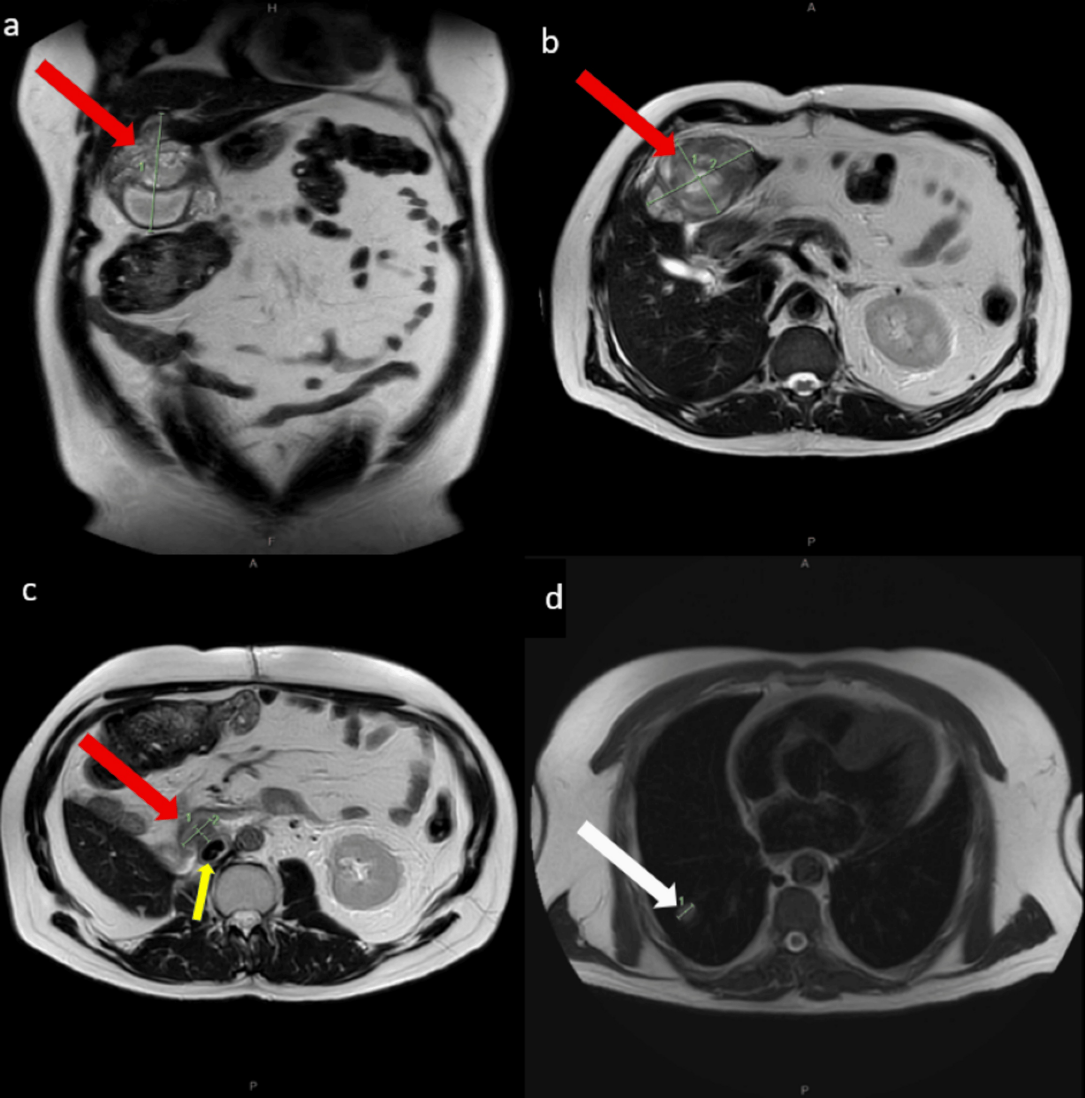
a-d showing MRI T2 phase six months post-operatively a: Coronal slice demonstrating a lesion within the inferior aspect of segment 3 of the liver (red arrow) measuring 8.4 x 5.9 x 9.5 cm (1 = 9.5cm). (b) Axial slice demonstrating a lesion within the inferior aspect of segment 3 of the liver (red arrow). (c) Axial slice showing suspicious recurrence within resection bed (red arrow) intimately related to reconstructed IVC (yellow arrow). (d) Axial slice showing right lung base lesion (white arrow) (1 = 1.36cm)

His case was discussed in the sarcoma multidisciplinary team meeting, and adjuvant chemotherapy and radiotherapy were recommended. He was commenced on consolidative radiation therapy to the right abdominopelvic retroperitoneum two months post-surgery. Given his recovering kidney function and severe systemic effects of chemotherapy, chemotherapy was not commenced, considering the uncertainty of chemotherapy benefit in his case. Six months post-surgery, his surveillance scans of MRI, PET, and CT demonstrated a 9.5 cm metastasis in the inferior aspect of segment III of the liver, possible recurrence within the resection bed intimately related to the anterior aspect of the reconstructed IVC graft, as well as bi-basal pulmonary metastasis with the largest within the right lower lobe measuring 1.4 cm (Figure 6).

Unfortunately, six months post-surgery, he presented with upper gastrointestinal bleed (UGIB), where he required blood transfusions and had a gastroscopy, which showed a large partially obstructing friable mass in the proximal duodenum (D2) as the likely source of bleed. He then received palliative radiotherapy to the duodenum as well as palliative chemotherapy of three-weekly doxorubicin with lipegfilgastrim with a non-curative intent.

He subsequently developed a duodenocaval fistula, which was treated with an emergency endovascular stent to control bleeding and antibiotics to treat IVC graft infection. He subsequently died 11 months post his surgery in a hospice under the care of the palliative team.

## Discussion

Retroperitoneal soft tissue sarcoma accounts for 12-15% of soft tissue sarcomas with an incidence rate of 2.7 per 1000000 people [1,5]. The types of retroperitoneal sarcoma are mesodermal, such as liposarcoma, leiomyosarcoma or RMS, neurogenic (neurofibroma) and extragonadal (teratoma) [1]. RMS has four subtypes, namely embryonal, alveolar, pleomorphic, and spindle cell or sclerosing, as described by the World Health Organization (WHO) [1,6]. Pleomorphic RMS are more commonly seen in adult patients (19.1%) and have the poorest prognosis [2-4]. Stout et al. first described pleomorphic RMS in 1946 [7]. WHO classifies pleomorphic RMS as a high-grade tumour which consists of undifferentiated, bizarre, atypical-looking, large multinucleated round, spindle, rhabdoid, and polygonal cells that show skeletal muscle differentiation with no evidence of embryonal or alveolar cells [1,6,8]. Pleomorphic RMS has an extremely complex karyotype, which explains its aggressive nature and poor outcome with conventional chemotherapy [6]. RMS tumour cells show positive desmin, myogenin, and MYOD1 immunohistochemically, as well as strong diffuse Wilms’ tumour 1 (WT1) cytoplasmic expression [8].

Pleomorphic RMS is most commonly diagnosed in elderly patients at a median age of 71.5 years, with an age range of 28.4 to 92.8 years. It is more common in males compared to females, with a ratio of 1.8:1. Most patients (71.1%) had localised disease at the time of diagnosis, with the primary tumour most commonly found in the extremities (51.1%). In patients with distant metastatic disease, the most commonly affected site was the lungs (76.9%) [6]. RMS has a 10-20% chance of metastasis from lymphatic or hematogenous dissemination [9]. RMS involving the retroperitoneum, as in our case, is extremely rare, accounting for 4% [4,10]. The most common retroperitoneal RMS is the embryonal subtype [8]. RMS does not usually result in nodal involvement, with less than 10% nodal involvement among patients who have localised disease [6].

Unfortunately, retroperitoneal sarcoma patients usually have a late presentation due to the presence of a large retroperitoneal space, which allows tumour expansion prior to resulting in symptoms which are often non-specific. A study showed that about 64% patients presented within six months of the start of symptoms, with the most usual presenting complaint being abdominal pain (50%), and the most common clinical finding was abdominal or pelvic mass (59%) [1,11]. Other symptoms are weight loss, as well as some other common nonspecific symptoms such as back pain, bloating, nausea, and vomiting due to the tumour's effect on adjacent organs. Other clinical findings seen were leg oedema [11].

Thorough investigation pre-operatively is essential due to the very complex retroperitoneal anatomy, as it provides an overview of the tumour size and the invasion of other organs or vessels, which aids in surgical planning [1]. Different modes of imaging and investigations can be used in the diagnosis of retroperitoneal sarcoma, which include ultrasound, CT, and ultrasound-guided biopsy (gold standard), as well as immunohistochemical studies, which are crucial for treatment planning [5]. In our case, the ultrasound-guided biopsy was not performed pre-operatively due to the high-risk of bleeding and need for surgery; however, the immunohistology studies done post-operatively, which were positive for desmin, guided towards the diagnosis of pleomorphic RMS [12]. Desmin can also be seen in leiomyosarcoma [1]. In situations where there is an uncertainty in diagnosis or pre-operative treatment plan, percutaneous core needle biopsy of retroperitoneal sarcomas (RPS) is supported by a few studies due to the very low chance of tumour seeding in the biopsy tract [2]. However, if there is an established diagnosis of RPS radiologically, surgery can be carried out without performing a biopsy [2].

Based on a study by Husnoo et al., 53% of retroperitoneal tumours had a diameter more than 10 cm, with a median diameter measuring 13.7 cm (ranging from 2.5 cm to 39 cm) at diagnosis [1]. The patient in this case report had a tumour mass measuring 9 cm. Complete tumour resection, adjuvant therapy, and re-resection after recurrence are very crucial as disease spread, even microscopically, would adversely affect prognosis. In a study by Garcia-Ortega et al., they emphasised complete resection of retroperitoneal sarcomas as a predictive factor for prognosis, and their study showed that patients with complete resection, R0, had a higher mean survival rate of 55.6 months compared to patients with incomplete resection, who had 28.1 months [13]. In this case, complete resection of the main primary tumour (IVC tumour) was performed; however, the superior IVC margin was occluded by luminal fibrin-thrombus containing probable admixed necrotic tumour cells, which could have contributed to metastatic disease resulting in poor prognosis with survival time of 11 months post-surgery.

The mainstay treatment for patients with pleomorphic RMS presenting with localised disease is wide surgical resection with the addition of radiation therapy either pre- or post-operatively, which may be curative in some patients. Distant relapse is common, as seen in our case, despite satisfactory surgical resection, especially in those with positive margins [6]. In order to achieve complete surgical excision, the resection of close-proximity organs is usually required [11]. The most preferred choice of surgical excision for RPS is transperitoneal, via midline or transverse incision, which permits easier access to the main blood supply and enables decision-making on tumour excision [11]. On the other hand, patients with pleomorphic RMS, especially those who have advanced disease, have no standard systemic therapy options; therefore, they are usually managed with various chemotherapy regimes, which range from single-agent doxorubicin to multi-agent paediatric chemotherapy regimes. Unfortunately, there is no strong agreement on the best systemic treatment option for pleomorphic RMS, as the majority of retrospective studies conducted have grouped various RMS subtypes together [6]. In general, pleomorphic RMS is known to be chemoresistant, and further studies on its chemosensitivity are ongoing [6]. For RPS patients with high- or intermediate-grade tumours who had R0/R1 resection, adjuvant radiotherapy is the available treatment of choice as there is a risk of local recurrence [2]. Among RMS patients with localised disease, 12.5% had positive margins on surgical resection, 53.8% had disease relapse with a relapse median time of 5.3 months and 57.7% required radiation therapy post-operatively [6]. A study by Kadam et al. recommends not to deliver adjuvant radiotherapy treatment due to morbidity and suggests re-resection if there is recurrence of tumour during post-operative surveillance [2].

With regard to further medical management, Noujaim et al. stated that adjuvant chemotherapy showed minimal success in adults with pleomorphic RMS, as only one out of 45 patients had a partial response post-paediatric-type chemotherapy regimen, which consisted of vincristine, actinomycin D, and cyclophosphamide. This is due to the highly complex karyotype of the pleomorphic RMS’s genetic component when compared to other types of RMS, such as alveolar and embryonal [6]. The influence of neoadjuvant chemotherapy seems equivocal, with limited evidence in relation to its potential success in the management of soft tissue sarcomata [14]. Pre-operative neoadjuvant chemoradiotherapy may aid in downstaging the tumour to allow for complete resection [14]. Single-agent doxorubicin is the most preferred first-line regimen for palliative chemotherapy [6]. In this case, the patient received palliative chemotherapy to dampen the progression of the disease and for symptomatic management, considering no available universally agreed consensus and very scarce literature on this condition due to its rarity.

Noujaim et al. again emphasised that broad surgical resection remains the primary predictive factor in the survival or prognosis of patients. Based on their study, adults with pleomorphic RMS have a poor prognosis, with median survival durations for patients with localised (71.1%) and metastatic disease (28.9%) of 12.8 months and 7.1 months, respectively. Besides that, pleomorphic RMS has a high relapse rate of 53.8% [6].

## Conclusions

Pleomorphic RMS is a very malignant soft-tissue sarcoma with a poor prognosis. Despite an aggressive, successful en bloc resection of this IVC tumour in curative intent, the patient unfortunately developed rapid metastatic disease recurrence, had a poor clinical outcome, and died within 11 months of his surgery, which explains the aggressive pathophysiology of RMS. Complete surgical resection together with radiotherapy, performed pre-operative or post-operative, remains the gold standard management for pleomorphic RMS patients with localised disease, which improves survival rates. Overall, pleomorphic RMS is considered resistant to standard chemotherapy; thus, more effective novel treatments should be discovered by studying its basic molecular pattern, which causes tumourigenesis that may contribute in better management plan for RMS patients in the future. It is important to consider standard or institutional surveillance protocol in patients with pleomorphic RMS due to the high risk of recurrence.
